# A rapid assessment of the quality of neonatal healthcare in Kilimanjaro region, northeast Tanzania

**DOI:** 10.1186/1471-2431-12-182

**Published:** 2012-11-21

**Authors:** Bernard Mbwele, Elizabeth Reddy, Hugh Reyburn

**Affiliations:** 1 Kilimanjaro Clinical Research Institute, P.O Box 2236, Moshi, Tanzania; 2 Duke University Dept. of Medicine; Division of Infectious Disease, Kilimanjaro Christian Medical Centre-Duke University Collaboration, P.O Box 3010, Moshi, Tanzania; 3 London school of Hygiene and Tropical Medicine, Disease Control Dept, Faculty of Infectious and Tropical Disease, London School of Hygiene and Tropical Medicine, Keppel St, London, WCIE 7HT, UK

## Abstract

**Background:**

While child mortality is declining in Africa there has been no evidence of a comparable reduction in neonatal mortality. The quality of inpatient neonatal care is likely a contributing factor but data from resource limited settings are few. The objective of this study was to assess the quality of neonatal care in the district hospitals of the Kilimanjaro region of Tanzania.

**Methods:**

Clinical records were reviewed for ill or premature neonates admitted to 13 inpatient health facilities in the Kilimanjaro region; staffing and equipment levels were also assessed.

**Results:**

Among the 82 neonates reviewed, key health information was missing from a substantial proportion of records: on maternal antenatal cards, blood group was recorded for 52 (63.4%) mothers, Rhesus (Rh) factor for 39 (47.6%), VDRL for 59 (71.9%) and HIV status for 77 (93.1%). From neonatal clinical records, heart rate was recorded for3 (3.7%) neonates, respiratory rate in 14, (17.1%) and temperature in 33 (40.2%). None of 13 facilities had a functioning premature unit despite calculated gestational age <36 weeks in 45.6% of evaluated neonates. Intravenous fluids and oxygen were available in 9 out of 13 of facilities, while antibiotics and essential basic equipment were available in more than two thirds. Medication dosing errors were common; under-dosage for ampicillin, gentamicin and cloxacillin was found in 44.0%, 37.9% and 50% of cases, respectively, while over-dosage was found in 20.0%, 24.2% and 19.9%, respectively. Physician or assistant physician staffing levels by the WHO indicator levels (WISN) were generally low.

**Conclusion:**

Key aspects of neonatal care were found to be poorly documented or incorrectly implemented in this appraisal of neonatal care in Kilimanjaro. Efforts towards quality assurance and enhanced motivation of staff may improve outcomes for this vulnerable group.

## Background

The World Health Organization estimates that 4 million children under 1 month of age die each year [1,2] and more than 90% of these deaths occur in developing countries [3,4]. Neonatal mortality rates have remained high despite a decline of infant mortality rates [5]. The highest neonatal mortality rates are documented in sub-Saharan Africa [1,6-9]. Tanzania is among the five countries in sub-Saharan Africa recording the highest neonatal mortality rates, [8,10] with birth asphyxia, prematurity and infection as prime causes, all of which are preventable [11].

The relative lack of progress in reducing neonatal mortality may be at least in part due to a gap between national or international standards of good care of neonates and the actual delivery of care health facilities [12-14]. However, the lack of quantitative and qualitative data, including an absence of stable and accurate vital registration systems [15-18] and retrieval systems, constitute constraints in making a useful judgment of the quality of care [10,19].

A study conducted in 2002 in the Kilimanjaro region of Tanzania found that only one-third of all inpatientdeaths were properly documented in the case notes, and the cause of death could only be determined in 38% of the these [20]. Most of the neonatal deaths in northern Tanzania are preventable, [21,22] and an audit of records in a Dar es Salaam teaching hospital showed quality neonatal care was often suboptimal even within the context of available resources [23].

This study aimed to provide an overview of the quality of neonatal health care in district hospitals and health centres offering obstetric services in the Kilimanjaro region in north-eastern Tanzania. We anticipate that the results may suggest priority areas for improvement of neonatal care nationally and in sub-Saharan Africa generally.

## Methods

The study was conducted in Kilimanjaro region of north-eastern Tanzania where data were collected from the northern zonal referral hospital and 13 referring peripheral hospitals by a purposive sampling technique. Of the 13 peripheral facilities included, 1 was the Kilimanjaro regional hospital, 4 were government-supported district hospitals, 6 were missionary-supported hospitals designated as district hospitals, and 2 were health centres with inpatient facilities servingurban Moshi’s two largest wards. Each health care facility was visited unannounced and data were collected in a single day or at most over two days.

In each facility, staffing numbers were recorded from nationally standardized system i.e. Health Management Information Systems (HMIS), used for collection of data regarding health care workers in Tanzania; these data were verified in discussion with senior administrative staff in each hospital. Vital registration records and antenatal cards, records care and referral records were reviewed at each facility as well as at the zonal referral hospital from 26^th^ November, 2010 to 25^th^ April, 2011.

**Figure 1:**
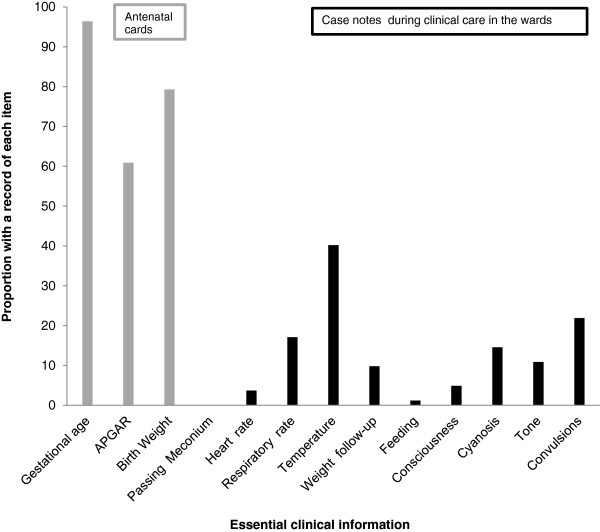
Presence of a record of essential clinical information as documented in 82 case notes of neonates who were admitted or retained in the ward for medical reasons.

Maternity and paediatric wards were inspected for the presence of basic supplies and equipment using a pre-prepared standard check-list. In addition, health workers were requested, again using a standardized list, to estimate the availability of essential supplies in terms of the number of months per year a supply was typically available. In each hospital the senior nurse or clinician who was present in each of the relevant paediatric or maternity wards was asked to identify any neonate less than or equal to 30 days of age currently on the ward who had been admitted or retained post-delivery for a medical problem, including prematurity. In each case, the record of the last menstrual period (LMP) as recorded on the antenatal card was used to calculate gestational age. The case notes were inspected and standard informationwas extracted regarding the presence or absence of a list of 13 features judged to be essential to the record of a sick neonate, as shown in Figure 1. A record of Rh factorand blood group, syphilis and HIV screening, APGAR score and birth weight were extracted from all antenatal cards.

### Data Management and Analysis

Data were single- entered in MS Access (Microsoft Corp, Redmond, VA). Original forms were consulted in the case of missing results or values out of expected ranges, and data were corrected as needed by the principal investigator and the data manager. Data were analysed using Stata-10 (Stata Corp, TX, USA).

Staffing levels were assessed usingworkload indicators of staffing need (WISN). The expected number of health workers was found by dividing a yearly total time required to attend all neonates by total time available per health care worker (standardised by World Health Organization manual) [24]. The time required to attend one neonate was derived from the facility based neonatal admissions [24,25].

Recordsof prescriptions for ampicillin, cloxacillin or gentamicin were extracted from case notes or prescription sheets and the dosages were assessed by reference to the WHO Emergency Triage Assessment and Treatment (ETAT) manual in the context of the neonate’s weight.

### Ethics

The study was approved by the Kilimanjaro Christian Medical University Ethics Committee. Written approval for the study was also received from the Kilimanjaro Regional Medical Officer. Written consent was obtained from the senior medical officer of each health facility for health workerinterviews, case notes assessments, checklists of supplies, photographs, and for results to be published.In addition, written consent was obtained from the guardians (mothers) of all enrolled neonates.

## Results

Summary details of thereferral records from the 13 peripheral hospitals and the zonal referral hospital are shown in Table 1 and Figure 1. Eighty-two sick neonateswere enrolled from the 13 peripheralfacilities and their case notes examined. The mean (standard deviation, SD) age at time of case note review was 8.7 (± 10.6) days.

**Table 1:** **Hospital data on neonatal births, deaths 1**^**st **^**January to 31**^**st **^**December 2010**

**Facility code**	**Facility referrals**	**Referrals arrived at a referral hospital**	**Live births**	**Deaths**	**Deaths / 1000 live births**
F01	44	140	4,506	14	3.1
F02	1	8	796	6	7.5
F03	9	23	794	1	1.3
F04	3	23	177	6	33.9
F05	24	15	303	2	6.6
F06	39	21	1,681	49	29.1
F07	1	22	997	2	2.0
F08	0	1	1,243	1	0.8
F09	1	7	4,016	5	1.3
F10	14	20	1,289	7	5.4
F11	3	2	525	1	1.9
F12	0	28	524	0	0
F 13	6	38	732	0	0
**Peripheral health facilities**	-	-	**17,583**	**94**	**5.3**
**Referral centre**	**N/A**	**1,155**	**4,074**	**273**	**67.01**

**Figure 2:**
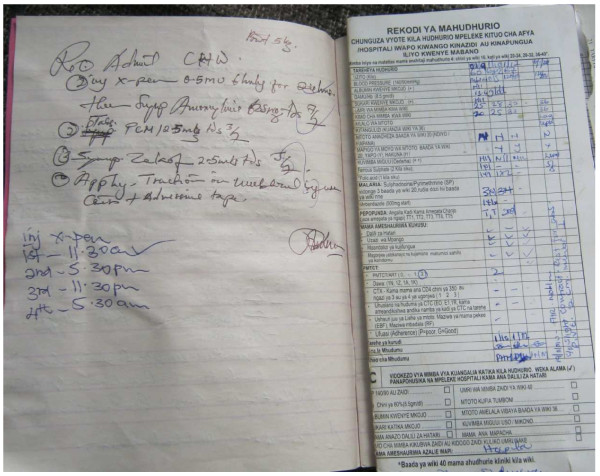
**Records of care in Daftari.** An example of primary school exercise books “Daftari” used in making records of neonatal care in one of the district hospital. On the right side is the sample of antenatal card we used to make assessment of records that are essential in neonatal care.

The locations of neonatal care within each facility (i.e. maternal, paediatric, or special dedicated wards) are described in Table 2. The neonatal chart notes, or records of care, were found in disparate locations in the different facilities: in 3 facilities neonatal case notes were found in local school exercise books “daftari” as shown in Figure 2, 8 hospitals detailed neonatal care in the mothers’ case notes, and 2 hospitals used standard medical files specific to the neonate. Antenatal cards were held by the mothers in 12 (92.3%) of the 13 facilities and were located in facility-based records in one hospital.

**Table 2:** **Staffing numbers and levels (by WISN) dedicated to neonatal care 1**^**st **^**January to 31**^**st **^**December 2010 in the peripheral facilities visited**

**Facility name and code**	**Location of neonatal care**	**Clinicians available for neonates**			**Nurses available for neonates**			**Nurse attendants for neonates**		
		**Per Week**	**AM shift**	**WISN**^**1**^	**Per Week**	**AM shift**	**WISN**^**1**^	**Per Week**	**AM shift**	**WISN**^**1**^
F01	Maternity	4	1	0.19	15	3	3.98	2	2	0.35
F02	Maternity	2	2	0.55	10	2	6.01	2	1	2.01
F03	Paediatric ward	5	1	1.37	12	3	18.09	13	2	13.07
F04	Maternity	2	1	2.46	7	2	47.33	5	1	22.56
F05	Maternity	2	2	1.44	10	2	39.50	5	1	13.18
F06	Maternity	2	2	0.26	8	2	5.70	4	1	1.90
F07	Maternity	4	2	0.87	12	2	14.40	1	1	0.80
F08	Maternity	3	1	0.53	7	2	6.74	4	2	2.57
F09	Maternity and Premature unit	2	1	0.11	15	2	0.60	6	1	0.40
F10	Maternity	3	1	0.51	4	2	3.71	5	2	3.10
F11	Maternity and Premature unit	4	2	1.66	6	2	4.56	2	1	3.04
F12	Maternity	3	1	1.57	3	2	8.56	1	1	1.90
F13	Labour ward	1	1	0.37	3	2	6.13	3	2	4.09

Footnote: WISN^1^ stands for WHO devised “Workload indicator for staff need”.

### Data extracted from antenatal cards

Antenatal cards of the all included neonates were assessed. The majority of mothers [59(71.9%)] received antenatal care at facilities different from where they ultimately delivered their infants. Overall, 72 (88.0%) mothershad attended an antenatal clinic at least three times (mean 2.95±1.3 visits) during their pregnancy and all 82 enrolled neonates were born in health care facilities.

Records for Rh factor and blood group were present on antenatal cards in 39 (47.6%) and 52 (63.4%) cases respectively while syphilis or HIV screening was recorded on 59 (71.9%) and 77 (93.1%) antenatal cards respectively. Among 77 mothers who had received counselling, testing, and/or services for prevention of maternal to child transmission of HIV (PMTCT), 64 (83.0%) were HIV negative, 12 (15.6%) were HIV positive, and 1 (1.3%) had declined testing.

Despite the 100% availability of APGAR score records in the delivery books at the included facilities, these records had been copied onto the antenatal cards in only 61.0% (50 records out of 82) cases. Birth weight records were available in 65 case notes (79.3%) despite all the infants having been born in a health care facility. The median birth weight of neonates whose files were evaluated was 3000 grams with an interquartile range of 2600–3350 grams.

### Clinical data on neonates present on the ward

**Figure 3:**
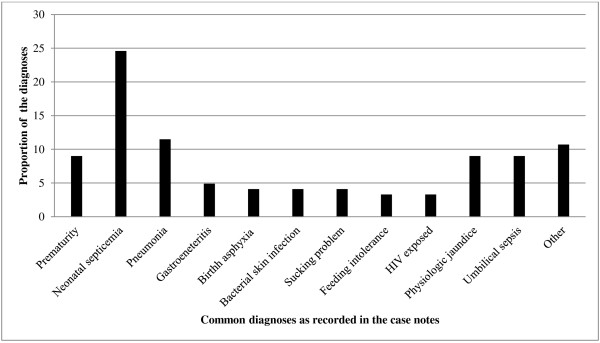
Diagnoses of admitted sick neonates present on the maternity or paediatric ward at the time of assessment.

Temperature was the most commonly recorded vital sign, found in 33 (40.2%) case notes. Two thirds (66.0%) of admitted neonates with records of temperature had a recording ≥38.0°C. Admitting diagnoses as abstracted from the case notes the 82 enrolled neonates are shown in Figure 3; 40 of the neonates had more than one admitting diagnosis. Prematurity, defined as a gestation age less than 36 weeks, was documented in only 11(9.0%) case notes while calculation from the antenatal card indicated that 36(45.6%) of 79 neonates with LMP data had a gestational age less than 36 weeks.

### Documentation and tracking referrals

Records of referrals were made in Ministry of Health forms and discordance between this record and documented arrival at the zonal referral hospital is shown in Table 1. In no cases did the number of referred and received cases match, and in one case 100% of the received patients were unaccounted for at the referring institution.

### Laboratory investigations

A record of full blood count was found in 4 (4.8%) cases, and haemoglobin wasdocumentedin 6 (7.3%) case notes. Nine (11%) case notes had a record of blood sugar while 22 cases had a record of altered consciousness or convulsions that would normally be an indication to measure blood sugar. There were no laboratory investigations for blood culture or bilirubin as none of the facilities had capacity to perform these tests. Overall, only 8 (9.8%) of case notes of the sick neonates in the district hospitals had record of any laboratory investigation.

### Prescriptions of oxygen, I.V fluids and Antibiotics

**Figure 4:**
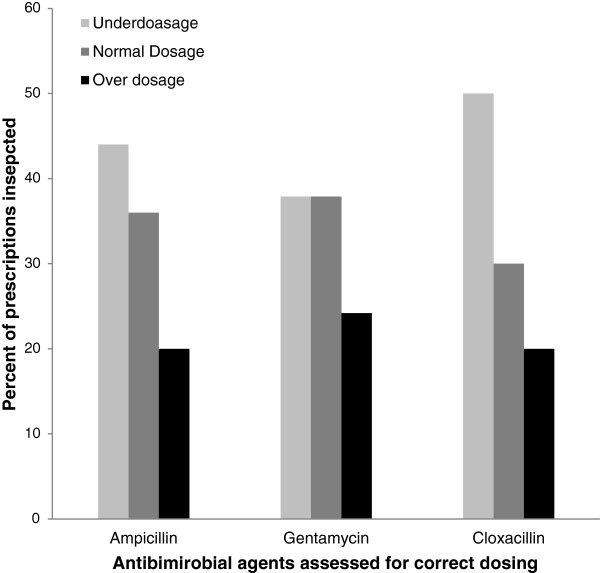
Assessment of prescriptions of doses given to sick neonates admitted in the district hospitals.

There were 16 neonates who, according to the record of care, met indications for oxygen therapy, but only 6 (37.5% of cases in which it was indicated) were documented to have received it. Only 8 neonates (9.7%) had documentation of IV fluids, whereas we estimate that 24 (29.3%) needed the intravenous fluid infusions due to clinical presentations of hypoglycaemia [glucose < 30 mg/dl] and/or a combination of high grade fever and inability to breast feed. Estimation of correct dosing of antimicrobial agents according to WHO guidelines is shown in Figure 4.

### Staffing of health workers

Staff availability is presented in Table 2. WISN for clinicians was 0.93 (ranging from 0.1 to 2.4), for nurses was 12.2 (ranging from 0.6 to 47.3), and for nurse attendants was 5.34 (ranging from 0.3 to 22.5).

### Supply of essential drugs and equipment

**Figure 5:**
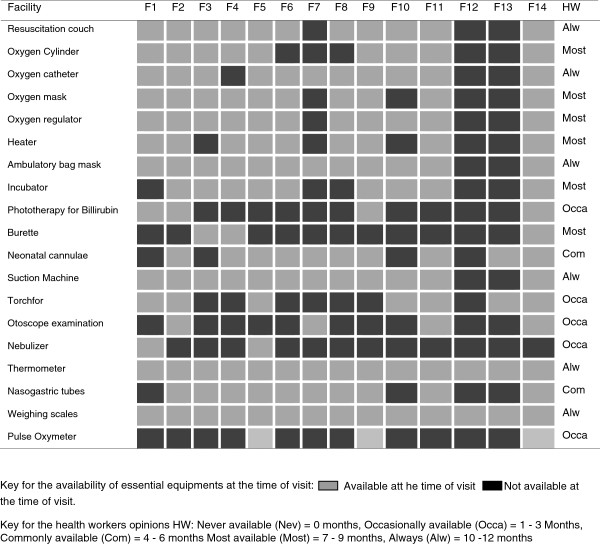
Availability of essential equipments as observed from the facility inspection on the day of survey and aggregated opinion of health staff on general availability of same items.

**Figure 6:**
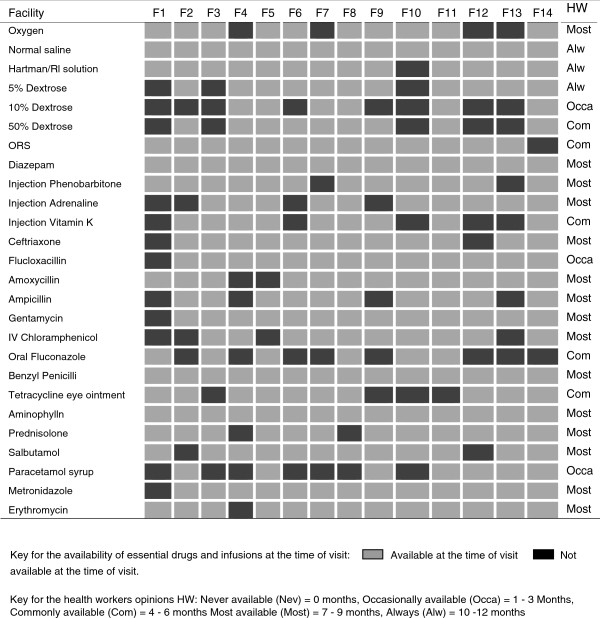
Availability of essential drugs and infusions as observed from the facility inspection on the day of survey and aggregated opinion of health staff on general availability of same items.

Basic equipment was commonly available at the district hospitals with a relatively poor availability at the two heath centres (Figure 5). Nine out of 13 peripheral hospitals (72.7%) in Kilimanjaro had visible stocks of essential drugs, oxygen and fluids to manage common neonatal infections and other disorders of mild severity such as transient tachypnoea of the new-born. These observations were subjectively well correlated with estimated availability in months per year reported by health workers interview (Figure 6).

## Discussion

Care of neonates in Kilimanjaro region is characterized by poor documentation and a lack of common systems for documentation across facilities; in addition many of our findings suggest substandard care even within the context of available resources. These findings are apparently similar to those in Kenya [26] and those found among older children in the same region [14]. While the neonatal mortality rates recorded at these facilities are lower than what might be expected given the conditions, the high mortality rate at the zonal referral hospital raises concern since many high risk women and neonates are transferred there.Substantial discrepancies noted between recorded referrals to thezonal referralhospital and records of received neonates at the zonal referral hospital demonstrate some of the challenges faced inhealth systems management, evaluation process and outcome of care.

One of our findings was the lack of a universal system for recording medical assessments and care provided to sick neonates, aside from the antenatal care card, which has very limited space for such evaluations. Neonatal care records were often difficult to locate and information provided within them was scant, with key diagnoses such as prematurity notably lacking in a substantial proportion of cases. Although absence of a record does not imply absence of examination, a poor record of care inevitably damages prospects for good continuity of care between staff and local systematic audits are difficult to perform. This is particularly important in neonatal care where clinical states change rapidly. Given the complexities that are unique to the care of neonates (high likelihood of hypothermia, hypoglycaemia, differences in “cut-off” for empiric antibiotics in the case of fever, need for different types of intravenous fluids), the availability of standard care forms which guide health care workers through the process of a typical neonatal admission could prove useful.

The fact that birth weight was missing from nearly one-fifth of universally filled antenatal cardsindicates poor motivation and/or oversight of clinical staff attending neonates delivered in health facilities. In addition to poor documentation, evidence of suboptimal care was prevalent. Incorrect dosing of antibiotics occurred in a substantial proportion of cases. Improbable diagnoses such as “gastroenteritis” were found on more than one occasion in chart notes, the latter being a highly unlikely diagnosis in neonates, especially in this setting where exclusive breastfeeding is almost universal through the neonatal period of one month; this is concerning since feeding intolerance and vomiting at this age could portend a much more serious diagnosis such as trachea-oesophageal fistula. The notably higher proportion of antenatal cards with HIV test results compared with other test results suggests that training, motivation, and efforts to make tests kits available on the ground can result in improved performance; there have been concerted efforts to promote prevention of maternal to child transmission in Kilimanjaro in recent years with highly successful results [27].

Missing laboratory tests, such as blood group and Rh factor in mothers and bilirubin in neonates is likely explained by lack of assay availability in many cases. The absence of anything beyond the most basic laboratory facilities at most of the hospitals in this study prevents proper diagnosis and management of key frequently encountered conditions. Since prematurity and infections contributed to most of the hospitalizations, availability of blood culture systems and measurement of full blood picture are critical to appropriate management of these neonates, but most often unavailable [28-30].

However, we showed that even when supplies and diagnostics are available, they are not utilized appropriately, as in the case of glucometers, oxygen and intravenous fluids. The relative lack of expertise in neonatal care almost certainly contributes to these challenges; WISN for nurses tended to be high, but for clinicians was much lower, indicating a need for more specialized training in this area [31]. Similar lack of expertise in managing antenatal care as well as in managing sick children in hospital has previously been documented in Tanzania [14,32-35]; therefore it may not be surprising that neonatal care faces similar challenges.

While declines in child mortality rates in sub-Saharan Africa are encouraging, these declines are likely to stagnate if quality of neonatal care is not addressed. Achieving MDG 4 remains a challenging task in rural Tanzania, [36,37] and sub-Saharan Africa [38]. Over the last 5 years declines in infant and child mortality rates have stagnated [39,40]. Our data demonstrate that improvements will need to cover several areas, but that better care may be delivered even within the confines of currently available staff and equipment if there are improvements in training, motivation and standard operating procedures/guidelines to address key issues in neonatal care.

### Limitations

Our study had several limitations. First, sampling of facilities was not random, though it included the entire group of district and designated district hospitals in the region. Second, the limitations of tools to measure the quality of care are well known; direct observation is likely to bias performance (the Hawthorne effect) and a record of care is not the same as care itself. That having been said, the fact that facility awareness and patient enrolment took place typically on the same day, bias related to altering quality of care because of direct observation is substantially reduced, which is a strength of the study. Third, resource constraints did not allow for a longer period of observation that would have produced larger numbers and more robust findings, however our data comprehensively assessed the most important aspects of care for common conditions found in neonates and the equipment available at each participating facility. Finally, this study did not evaluate the care of healthy neonates with no identified problems and did include neonates born at home or in smaller health care facilities, such as dispensaries. We assumed infants with high-levelcare needs were referred to the zonal referral hospital; the infants in this study reflected a level of mild-to-moderate illness severity for which we expected that their care generally should be able to be managed at a district hospital level.

## Conclusion

Documentation of care for sick neonates hospitalized in peripheral health facilities in Kilimanjaro demonstrates significant deficits in areas which could have major health implications, not only for infant outcomes but also for program assessment and planning. Lack of regular auditing, few highly trained staff,and low availability of equipment and laboratory facilities all contribute to these challenges.

### Recommendations

There is a need to enhance neonatal-specific training for hospital staff, and introduce a standard clinical record form for sick neonates admitted to hospital facilities.

## Competing interests

The study was funded by the Tanzanian Ministry of Health and Social Welfare as the first authors’ prerequisite study for the completion of MSc Clinical Research at Kilimanjaro Christian Medical University. Additional working support and expertise was given by volunteers who were medical personnel and university students with data entry experience. There have been noreimbursements, fees, funding, nor salary from any organization that depends on or influence the results of this study. The author does not hold any stocks or shares in an organization that may in any way might be affected by this publication.

## Authors’ contribution

BM developed a concept of research work, proposal development, data collection, database development, analysis, report writing and writing of the manuscript. HR was the external supervisor and the organiser who provided technical guidance of proposal development, data collection and analysis. ER assisted in the planning of data extraction tools and in writing of the manuscript. All authors read and approved the final manuscript.

## Authors’ information

BM is a Tanzanian medical doctor and clinical researcher at Kilimanjaro Clinical Research Institute of Kilimanjaro Christian Medical Centre, Moshi Tanzania. HR is faculty at the London school of Hygiene and Tropical Medicine and he is a project leader at the Joint Malaria Programme in Moshi, Tanzania. He was the organiser and the overall supervisor for proposal writing and report designs. ER is a paediatrician, internist and researcher who directs collaborative research programs between Duke University and Kilimanjaro Christian Medical Centre.

## Pre-publication history

The pre-publication history for this paper can be accessed here:

http://www.biomedcentral.com/1471-2431/12/182/prepub
